# CT-derived extracellular volume fraction as a predictive marker for postoperative recurrence in pStage II–III gastric cancer

**DOI:** 10.1007/s00330-025-11765-0

**Published:** 2025-06-18

**Authors:** Yusuke Nishimuta, Daisuke Tsurumaru, Nobuhiro Fujita, Satohiro Kai, Junki Maehara, Yasuhiro Ushijima, Eiji Oki, Kousei Ishigami

**Affiliations:** 1 Department of Clinical Radiology, Graduate School of Medical Sciences, Kyushu University, Higashi-Ku, Japan; 2 Department of Endoscopic Diagnostics and Therapeutics, Kyushu University Hospital, Higashi-Ku, Japan; 3 Department of Anatomic Pathology, Graduate School of Medical Sciences, Kyushu University, Higashi-Ku, Japan; 4 Department of Surgery and Science, Graduate School of Medical Sciences, Kyushu University, Higashi-Ku, Japan

**Keywords:** Stomach neoplasms, Extracellular space, Multidetector computed tomography, Treatment outcome

## Abstract

**Objective:**

This study assessed the prognostic value of CT-derived extracellular volume fraction (CT-ECV) in predicting postoperative recurrence in patients with pathological stage (pStage) II–III gastric cancer (GC).

**Materials and methods:**

A retrospective analysis was conducted on 112 patients with pathologically confirmed pStage II–III gastric adenocarcinoma who underwent preoperative triphasic contrast-enhanced CT and curative gastrectomy without neoadjuvant therapy. The relationship between preoperative CT-ECV and recurrence risk was evaluated using comprehensive imaging and clinicopathological data. The optimal CT-ECV threshold for recurrence prediction was determined using receiver operating characteristic (ROC) curve analysis. Disease-free survival (DFS) was assessed using Kaplan–Meier and Cox regression analyses.

**Results:**

The mean CT-ECV was 56.4 ± 16.7%. Patients with recurrence (*n* = 28) had significantly higher CT-ECV values than those without recurrence (*n* = 84) (65.3 ± 14.3% vs 53.5 ± 16.5%; *p* < 0.001). The optimal CT-ECV cutoff for recurrence prediction was ≥ 56.9%, with an area under the curve of 0.71 (sensitivity 82.1%, specificity 61.9%). Multivariate analysis revealed that high CT-ECV was independently associated with worse DFS (HR: 5.93; 95% CI: 1.77–19.86; *p* = 0.004). Patients with high CT-ECV had significantly lower DFS rates compared to those with low CT-ECV (5-year DFS rate: 49.9% vs 93.7%; *p* < 0.001).

**Conclusion:**

High CT-ECV values correlate with increased recurrence risk and shorter DFS in pStage II–III GC, highlighting its potential as a predictive imaging biomarker for preoperative risk stratification.

**Key Points:**

***Question*** *Identifying prognostic markers is crucial for improving outcomes in stage II–III GC with high recurrence rates post-treatment*.

***Findings*** *High preoperative CT-ECV values are independently associated with increased recurrence risk and reduced DFS in pStage II–III GC*.

***Clinical relevance*** *CT-ECV can facilitate personalised treatment strategies and potentially improve patient management and outcomes*.

**Graphical Abstract:**

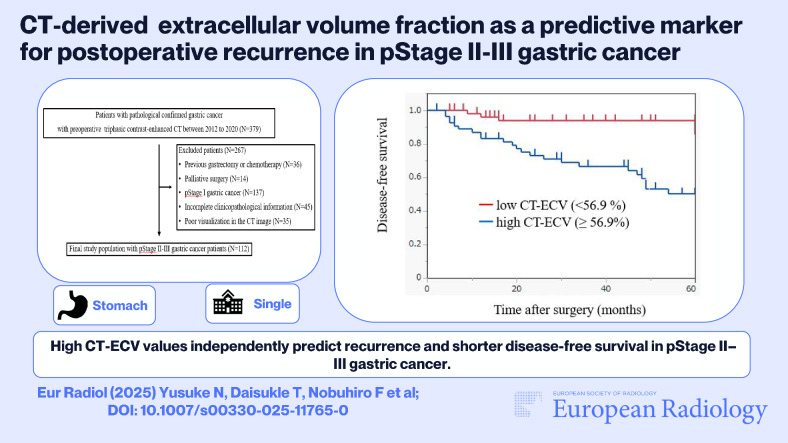

## Introduction

Gastric cancer (GC) is one of the most common malignant tumours of the gastrointestinal tract. Although the prognosis of patients with GC has improved due to advances in diagnostic techniques and intraoperative and postoperative care, GC remains the fourth leading cause of cancer-related deaths worldwide [1]. While patients with stage I GC who undergo endoscopic or surgical resection have excellent prognoses [2, 3], those with stage II–III GC face a high recurrence rate, even after radical resection and adjuvant chemotherapy [4, 5]. Effective treatment selection for these patients requires preoperative determination of tumour characteristics. Therefore, there is a critical need to identify novel prognostic markers, alongside current pathological staging, to optimise patient management and improve oncological outcomes.

Research interest in the tumour microenvironment and its role in tumourigenesis has recently grown. Several pathological and molecular biology studies have reported that the tumour stroma, which comprises immune cells, cancer-associated fibroblasts (CAFs), capillaries, and the extracellular matrix (ECM) surrounding cancer cells, influences tumour behaviour, including growth, dissemination, angiogenesis, immune evasion, and chemotherapy resistance [6–11]. Although the underlying biological mechanisms remain to be elucidated, early evidence suggests that stroma-rich tumours have a negative prognostic impact [12]. In GC, patients with a high proportion of stroma have poorer prognoses than those with a low proportion [13, 14]. However, because the tumour stroma in GCs cannot be accurately assessed through endoscopic biopsy specimens and can only be evaluated postoperatively, it remains challenging to incorporate this information into preoperative treatment planning.

The extracellular volume fraction (ECV), comprising the intravascular space and extravascular ECVs, can be quantified using equilibrium-enhanced computed tomography (CT) or magnetic resonance (MR) imaging. Several studies have shown that ECV values obtained from CT and MR imaging correspond with the extracellular stroma observed in biopsy specimens and can thus be used to quantify the extracellular stroma in cardiac or liver tissues [15–18]. Furthermore, CT/MR-derived ECV has been recognised as a valuable imaging marker for various solid tumour types [19–24]. To our knowledge, studies on the utility of ECV as an imaging biomarker in GC patients are limited [25, 26]. We hypothesised that preoperative ECV values derived from equilibrium contrast-enhanced CT (CT-ECV) could predict postoperative outcomes in patients with stage II–III GC. This study aims to explore the relationship between preoperative CT-ECV and recurrence risk in patients with pathological stage (pStage) II–III GC following curative gastrectomy.

## Materials and methods

### Ethics

This study was approved by the appropriate ethics committee and conducted in accordance with the principles of the Declaration of Helsinki. The requirement for informed consent was waived due to the retrospective nature of the study. Data were acquired in compliance with all applicable Health Insurance Portability and Accountability Act (HIPAA) regulations, and all methods were performed in line with relevant guidelines and regulations. Data were anonymised prior to analysis.

### Study design and patients

**Fig. 1:**
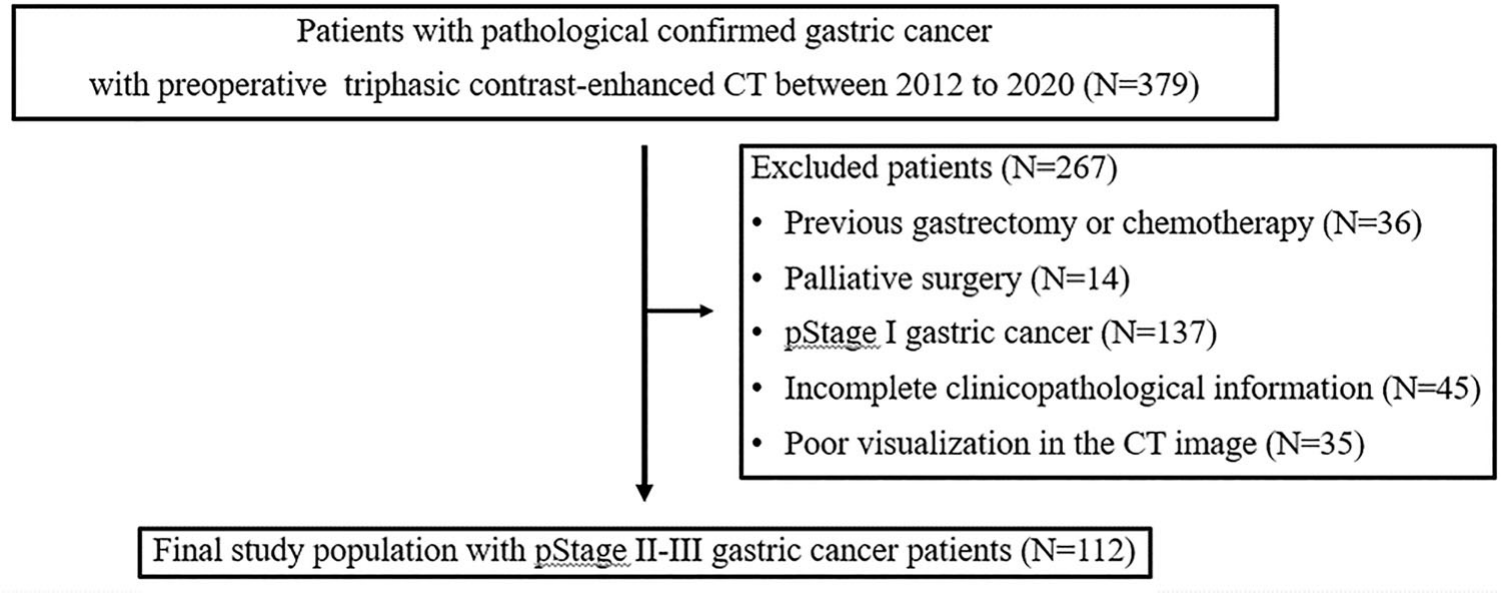
Flow chart of patient selection

This retrospective study initially evaluated 379 patients who underwent gastrectomy for GC within one month of triphasic contrast-enhanced CT (CECT) examination between April 2012 and March 2020. The exclusion criteria were as follows: (1) previous gastrectomy or chemotherapy (*N* = 36), (2) palliative surgery (*N* = 14), (3) pStage I GC (*N* = 137), (4) incomplete medical records and survival data (*N* = 45), and (5) poor CT image quality due to motion artefacts or unsatisfactory gastric distension (*N* = 35). Consequently, a total of 112 consecutive GC patients were enroled in this study. All patients underwent R0 gastrectomy with systematic lymphadenectomy, performed in accordance with the Japanese GC Treatment Guidelines [27], and were diagnosed with stage II or III gastric adenocarcinoma based on the 8th edition of the TNM Classification of Malignant Tumours. Figure 1 displays the patient selection flowchart. Among the included patients, 54 (48.2%) had pStage II GC, while 58 (51.8%) had pStage III GC.

### Patient management

All patients underwent gastrectomy with lymphadenectomy in accordance with the Japanese GC Treatment Guidelines [27]. All gastric adenocarcinomas were confirmed pathologically postoperatively. None of the patients received preoperative chemotherapy or radiotherapy. All patients were prescribed postoperative adjuvant chemotherapy with S-1 (an oral fluoropyrimidine derivative) or an oxaliplatin-based combination, unless contraindicated by the patient’s condition. In this study, curative resection was defined as the complete removal of the gastric tumour, including any metastatic sites, with no remaining macroscopic tumours. The patients were followed postoperatively for 4–60 months (median, 50 months), with CT scans every six months to facilitate early detection of recurrence.

### Data collection

The following demographic and clinicopathological data were collected from the medical records: age at diagnosis, sex, survival status at last follow-up, serum haematocrit level, and serum levels of tumour markers carbohydrate antigen (CA) 19-9 and carcinoembryonic antigen (CEA) within one week of CT examination. Additional data extracted from pathological reports included tumour differentiation, depth of tumour invasion (T stage), lymph node metastasis (N stage), lymphatic permeation, vascular invasion, and perineural invasion, as per the 8th edition of the TNM classification of malignant tumours.

### CT image acquisition

All CT examinations were performed using a 320-detector-row CT scanner (Aquilion One, Canon Medical Systems Corporation). Imaging parameters were as follows: tube voltage, 120 kVp; tube current, 200 mA; rotation time, 0.5 s; pitch, 0.797; detector row configuration, 64 × 0.625 mm; and image reconstruction thickness, 1 mm. To achieve gastric pouch distension, patients fasted overnight and ingested 5.25 g of an effervescent agent (Baros Effervescent Granules-S; Horii Pharmaceutical Industries) with a small amount of water immediately before scanning. After acquiring unenhanced CT images, multiphasic CT images were obtained at 40 s (arterial phase), 70 s (portal phase), and 240 s (equilibrium phase) following the infusion of 2 mL/kg of non-ionic contrast material (Iopamiron370; Bayer Health Care) at a rate of 3 mL/s. Patients were positioned supine throughout the imaging. All CT image datasets were transferred to a commercially available workstation equipped with image reconstruction software (Synapse Vincent; Fujifilm) for analysis.

### Image analysis

All CT images were independently reviewed by two radiologists, with 18 years and 15 years of experience in gastrointestinal radiology, respectively, who did not participate in the clinical reading sessions. Both observers were blinded to clinical information, including treatment details, tumour marker levels, and patient outcomes. Regions of interest (ROIs) were carefully delineated manually on the axial slice showing the largest area of the lesion and the aorta at the GC level on both unenhanced and equilibrium phase-enhanced CTs. This process referenced the arterial and venous phases of enhanced CT imaging as necessary, with care taken to avoid artefacts or necrotic/cystic changes within the ROIs. Measurements from the two observers were averaged to represent each ROI. The ECV of each GC tumour was calculated using the following equation: ECV (%) = (1 − haematocrit) × (ΔHUtumour/ΔHUaorta) × 100, where ΔHUtumour and ΔHUaorta represent the difference in Hounsfield units (HU) in the equilibrium phase minus the HU values in the tumour and aorta prior to contrast administration, respectively.

### Statistical analysis

Interobserver agreement for CT-ECV was evaluated by calculating the intraclass correlation coefficient (ICC), with ICC values > 0.90 indicating excellent agreement [28]. Clinicopathological characteristics were compared between the recurrence and recurrence-free groups using the χ² test or Fisher’s exact test for categorical variables and the two-sample *t*-test or Mann–Whitney *U*-test for continuous variables. The optimal CT-ECV cutoff value for predicting postoperative recurrence was determined using receiver operating characteristic (ROC) curve analysis with the Youden index. Correlations between these factors and disease-free survival (DFS) were analysed using the Cox proportional hazards model. DFS was defined as the interval from surgery to tumour relapse, either locally or distantly. Given the limited sample size, multivariate analysis was performed using only significant clinicopathological factors identified in univariate analysis. To minimise overfitting [29], lymph vascular perineural invasion (LVPI) was used as a parameter, combining lymphatic invasion, venous invasion, and perineural invasion rather than treating each factor as an independent variable. DFS was estimated according to the independent factors identified in multivariate analysis using the Kaplan–Meier method and compared between groups with the log-rank test. All statistical analyses were conducted using the open-source R platform, with statistical significance set at *p* < 0.05.

## Results

### Patient characteristics

There were 66 males and 46 females, with a median patient age of 69 years (range, 32–98 years). The patient characteristics are presented in Table 1. The mean CT-ECV was 56.4 ± 16.7%. The inter-observer agreement between the two readers for CT-ECV measurement was excellent, with an ICC of 0.93 (95% CI: 0.89–0.96). Overall, 21 patients (18.8%) had differentiated subtypes (well or moderately differentiated adenocarcinoma), while 91 patients (81.2%) had undifferentiated subtypes (poorly differentiated adenocarcinoma or signet-ring cell carcinoma). Regarding the pT stage, 4 (3.6%), 22 (19.6%), 54 (48.2%), and 32 (28.6%) patients had pT1, pT2, pT3, and pT4 disease, respectively. For the pN stage, 22 (19.6%), 26 (23.2%), 32 (28.6%), and 32 (28.6%) patients had pN0, pN1, pN2, and pN3 stage disease, respectively. LVPI was observed in 79 (70.5%) patients. At the time of analysis, 28 patients (25.0%) had experienced postoperative recurrence: 17 patients had peritoneal dissemination, 6 had liver metastases, 2 had lung metastases, and 3 had local recurrence with lymph node metastases.

**Table 1:** Patient’s characteristics

Valuable	Total (*N* = 112)	Recurrence-free group (*N* = 84)	Recurrence group (*N* = 28)	*p* value
Age (years)	69 (32–98)	70 (32–98)	68 (35–81)	0.155
Sex (male/female)	66/46	51/33	15/13	0.515
CEA (ng/mL)	1.9 (0.2–102.0)	1.9 (0.2–102.0)	1.9 (0.5–65.3)	0.824
CA 19-9 (U/mL)	10.2 (0.6–259.6)	10.3 (0.6–104.7)	7.3 (0.6–259.6)	0.313
Tumour location (upper/middle/lower)	30/53/29	21/40/23	9/14/6	0.884
pT stage (1/2/3/4)	4/22/54/32	3/19/43/19	1/3/11/13	0.093
pN stage (0/1/2/3)	22/26/32/32	22/24/27/11	0/2/5/21	**< 0.001**
Differentiation (differentiated/undifferentiated)	21/91	18/66	3/25	0.271
LVPI (negative/positive)	33/79	29/55	4/24	0.055
CT-ECV (%)	56.4 ± 16.7	53.5 ± 16.5	65.3 ± 14.3	**0.001**

The bold values are indicate statistically significant differences *p* < 0.05

*CEA* carcinoembryonic antigen, *CA19-9* carbohydrate antigen 19-9, *LVPI* lymph vascular perineural invasion, *CT-ECV* contrast-enhanced computed tomography derived extracellular volume fraction

### Differences in clinicopathological parameters and CT-ECV between the recurrence-free and recurrence groups

**Fig. 2:**
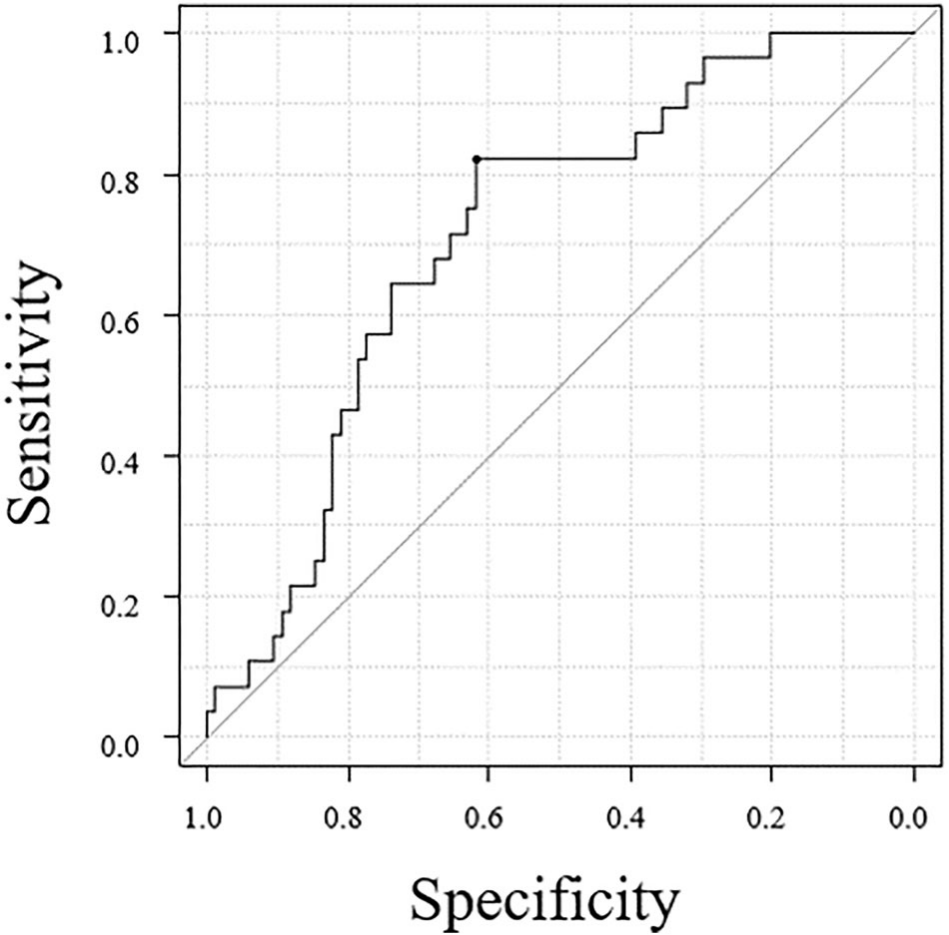
ROC curve analysis. Determination of the feasible cut-off point of the CT-ECV to predict postoperative recurrence, with a ROC curve. The area under the curve was 0.71 (sensitivity 82.1%, specificity 61.9%)

Compared with the recurrence-free group, the recurrence group had more advanced pN stages and higher CT-ECV values. The recurrence group showed significantly higher CT-ECV values than the recurrence-free group (65.3 ± 14.3% vs 53.5 ± 16.5%; *p* = 0.001). Other clinicopathological factors did not significantly differ between the two groups (Table 1). The optimal cutoff value of CT-ECV for identifying postoperative recurrence was ≥ 56.9%, with a sensitivity of 82.1%, specificity of 61.9%, and an area under the ROC curve of 0.71 (Fig. 2).

### Univariate and multivariate analysis on the DFS of GC patients after curative surgery

Univariate analysis indicated that pN2-3 (HR = 10.21, 95% CI: 2.42–43.06, *p* = 0.002), LVPI (HR = 3.20, 95% CI: 1.11–9.23, *p* = 0.032), and high CT-ECV (HR = 5.03, 95% CI: 1.91–13.23, *p* = 0.001) were associated with poorer DFS. Multivariate analysis confirmed that pN2-3 (HR = 6.83, 95% CI: 1.60–29.14, *p* = 0.001) and high CT-ECV (HR = 4.60, 95% CI: 1.72–12.31, *p* = 0.002) were independent predictors of poor DFS (Table 2).

**Table 2:** Univariate and multivariate analysis on progression-free survival of GC patients after curative surgery (*N* = 112)

Factor			Univariate analysis			Multivariate analysis		
			HR	95% CI	*p*	HR	96% CI	*p*
Age		No						
	< 65	28	Reference					
	≥ 65	57	0.84	0.34–2.08	0.7			
Sex								
	Male	66	Reference					
	Female	46	1.12	0.53–2.35	0.771			
CEA								
	< 3.2 ng/mL	80	Reference					
	≥ 3.2 ng/mL	32	1.12	0.49–2.53	0.796			
CA19-9								
	< 37.0 U/mL	101	Reference					
	≥ 37.0 U/mL	11	0.9	0.21–3.80	0.887			
Differentiation								
	Differentiated	21	Reference					
	Undifferentiated	91	2.37	0.71–7.85	0.159			
Location								
	Middle or Lower	82	Reference					
	Upper	30	1.36	0.91–2.02	0.131			
pT stage								
	pT1–2	4	Reference					
	pT3–4	108	2.34	0.81–6.74	0.117			
pN stage								
	pN0–1	48	Reference			Reference		
	pN2–3	64	10.21	2.42–43.06	**0.002**	6.83	1.60–29.14	**0.009**
LVPI								
	Negative	33	Reference			Reference		
	Positive	79	3.2	1.11–9.23	**0.032**	2.76	0.94–8.08	0.064
CT-ECV								
	< 56.9%	57	Reference			Reference		
	≥ 56.9%	55	5.03	1.91–13.23	**0.001**	4.6	1.72–12.31	**0.002**

The bold values are indicate statistically significant differences *p* < 0.05

*HR* hazard ratio, *CI* confidence interval, *CEA* carcinoembryonic antigen, *CA19-9* carbohydrate antigen 19-9, *LVPI* lymph vascular perineural invasion, *CT-ECV* contrast-enhanced computed tomography derived extracellular volume fraction

### Stratified DFS analysis using CT-ECV

**Fig. 3:**
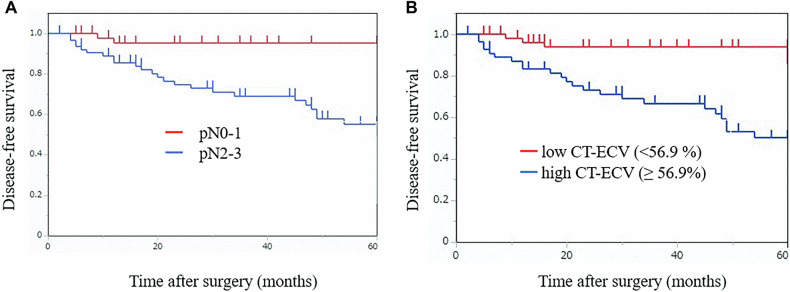
Kaplan–Meier survival curves of the DFS based on pN stage (**A**) and CT-ECV (**B**)

**Fig. 4:**
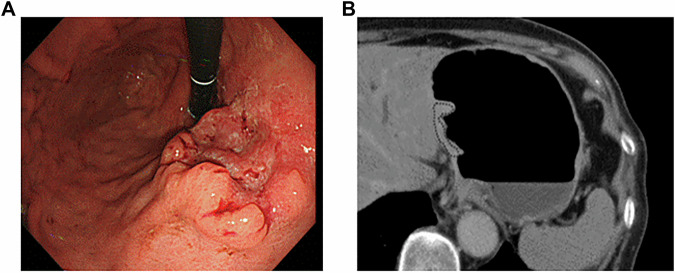
The 70 F pT2N1M0 pStage IIA. Advanced GC in groups of low CT-ECV. **A** Optical endoscopy shows type 3 cancer in the lesser curvature of the gastric body. **B** The CT-ECV of the lesion was 42.5%. The patient was relapse-free at 5 years after surgery and later

**Fig. 5:**
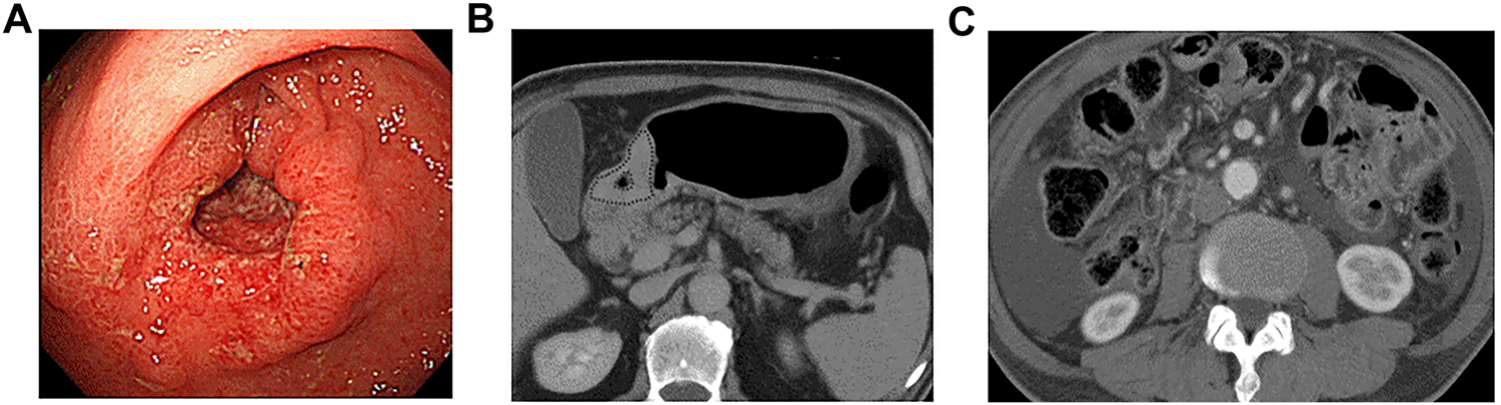
Case 2 62 M pT4aN3M0 pStageIIIc. Advanced GC in groups of high CT-ECV. **A** Optical endoscopy shows type 3 cancer in the gastric antrum. **B** The CT-ECV of the lesion was 67.8%. **C** Peritoneal dissemination occurred 2 years after surgery

Survival analysis demonstrated that the 5-year DFS rate was significantly lower in the pN2-3 group (54.9%, 95% CI: 40.1–67.6) compared to the pN0-1 group (95.2%, 95% CI: 82.1–98.8) (*p* < 0.001, Fig. 3A). Similarly, the high CT-ECV group had a significantly lower 5-year DFS rate (49.9%, 95% CI: 33.9–63.9) than the low CT-ECV group (93.7%, 95% CI: 81.5–97.9) (*p* < 0.001, Fig. 3B). Figures 4 and 5 display CT images of representative patients in the recurrence-free and recurrence groups, respectively.

## Discussion

Preoperative assessment of tumour aggressiveness using non-invasive imaging is crucial for reducing the risk of postoperative recurrence in GC. This study demonstrates that patients with stage II–III GC who develop recurrence have significantly higher CT-ECV values than those who do not. Notably, a high CT-ECV of ≥ 56.9% independently predicts poor DFS.

The tumour microenvironment, particularly the stromal component, plays a crucial role in GC progression. CAFs and ECM remodelling contribute to tumour growth, invasion, and treatment resistance by increasing fibrous stromal components [30]. Proteases secreted by cancer and mesenchymal cells lead to epithelial tissue disruption and ECM remodelling [31]. Such ECM changes result in poor vascularisation and hypoxia, impeding effective drug delivery [32]. These conditions are associated with more aggressive tumours and poorer prognoses, potentially explaining the worse outcomes in patients with high CT-ECV.

The ECV represents the sum of the intravascular space and extracellular extravascular volume fractions, reflecting both microvascular density and stromal fibrosis. Thus, it provides a comprehensive representation of the tumour microenvironment [19, 20]. Previous studies have demonstrated the diagnostic and prognostic value of CT-ECV in various tumours. For instance, Takumi et al explored the diagnostic utility of CT-ECV in thymic epithelial tumours and found that high CT-ECV was indicative of thymic carcinoma [21]. Fujita et al observed that a lower CT-ECV in pancreatic ductal adenocarcinoma (PDAC) correlated with a better response to neoadjuvant chemotherapy, while higher values suggested a poorer outcome [22]. These findings indicate that a higher ECV fraction suggests a greater malignancy potential, consistent with previous findings that stroma-rich tumours derive significant benefit from their tumour growth-promoting properties. In contrast, Fukukura et al reported a positive correlation between low ECV and poor prognosis in patients with unresectable or stage IV PDAC undergoing chemotherapy [23, 24]. These discrepancies may stem from differences in study populations, cancer types, stages, and treatment regimens, warranting further investigation.

Texture analysis has gained attention in GC management as it provides insights into tumour heterogeneity through pixel variation analysis [33]. Texture analysis provides information on the intrinsic variation of pixels within a tumour, serving as a non-invasive method for assessing tumour heterogeneity and potentially reflecting intrinsic aggressive biology or treatment resistance in a tumour. Giganti et al [34] demonstrated the prognostic relevance of CT texture analysis in GC by linking it with overall survival. However, its clinical applicability remains limited due to the need for complex processing. In contrast, the equilibrium CECT approach used in this study offers enhanced clinical versatility. CT-ECV is simple, reproducible, and integrates seamlessly into routine radiological assessments, allowing surgeons to optimise surgical plans by ensuring wider resection margins. Furthermore, patients with elevated CT-ECV may benefit from stricter postoperative monitoring, enabling early recurrence detection and intervention.

In Western countries, neoadjuvant or perioperative chemotherapy is standard for locally advanced GC [35]. Although our study focused on patients receiving adjuvant chemotherapy, CT-ECV may be applicable in other clinical contexts. Since pre-treatment CT-ECV reflects fundamental tumour microenvironment characteristics, it could be valuable in evaluating response prediction before neoadjuvant therapy. Chen et al reported that far-advanced GC with lower CT-ECV responded better to preoperative immunochemotherapy [26], suggesting that non-invasive imaging biomarkers like CT-ECV may be useful for therapy response evaluation.

This study also identified pN stage as an independent prognostic factor for DFS, aligning with previous reports [36, 37]. However, accurate preoperative assessment of lymph node metastasis remains challenging due to imaging limitations [38, 39]. While CT-ECV can be assessed preoperatively, further research is needed to explore the prognostic value of combining CT-ECV with pN stage. Given the limited sample size, developing a diagnostic model incorporating these factors remains a future goal.

The present study has several limitations. Firstly, the retrospective nature of the study, the relatively small patient sample size, and the wide variability in the observation period limit the generalizability of the results and highlight the need for large-scale prospective validation studies to confirm these findings. Secondly, the inclusion of only patients with stage II–III GC may have introduced selection bias. Patients with stage IV disease were excluded due to ongoing debates regarding the benefits of resection in these patients, given their uncertain short- and long-term outcomes. Similarly, patients with stage I disease were excluded to minimise treatment bias, as some may have undergone endoscopic or local resections without subsequent postoperative therapies. Thirdly, ROIs were delineated without accounting for tumour heterogeneity, and the largest lesion slice was assessed only once by each reader, potentially leading to sampling errors and discrepancies between imaging and histopathology. Moreover, the use of non-contrast and equilibrium-phase CT images for ECV calculations may have introduced positional discrepancies, reducing measurement accuracy. Future studies employing volumetric analysis and dual-energy CT could address these concerns. Fourthly, intratumoural angiogenesis and fibrosis were not assessed histologically, leaving the association between ECV and the tumour stroma speculative. Finally, the correlation between CT-ECV and overall survival was not assessed due to logistical challenges. Several patients with recurrence were transferred to other hospitals for supportive care due to disease progression, limiting our ability to monitor these patients and gather comprehensive information. Additionally, therapeutic strategies for managing postoperative recurrence varied among patients.

In conclusion, high CT-ECV values were found to be associated with postoperative recurrence and serve as an independent predictor of DFS following curative surgery in patients with pStage II–III GC. These findings support CT-ECV as a potential prognostic imaging marker in this patient cohort. Further large-scale studies are required to confirm whether incorporating CT-ECV into preoperative evaluation protocols can enhance personalised treatment strategies and ultimately improve patient outcomes.

## Abbreviations

CAFs: Cancer-associated fibroblasts
CT-ECV: CT-derived extracellular volume fraction
DFS: Disease-free survival
ECM: Extracellular matrix
ECV: Extracellular volume fraction
GC: Gastric cancer
LVPI: Lymph vascular perineural invasion
PDAC: Pancreatic ductal adenocarcinoma
pStage: Pathological stage
ROC: Receiver operating characteristic
ROIs: Regions of interest
