# Correction: CT-derived extracellular volume fraction as a predictive marker for postoperative recurrence in pStage II–III gastric cancer

**DOI:** 10.1007/s00330-025-12037-7

**Published:** 2025-10-17

**Authors:** Yusuke Nishimuta, Daisuke Tsurumaru, Nobuhiro Fujita, Satohiro Kai, Junki Maehara, Yasuhiro Ushijima, Eiji Oki, Kousei Ishigami

**Affiliations:** 1 Department of Clinical Radiology, Graduate School of Medical Sciences, Kyushu University, Higashi-Ku, Japan; 2 Department of Endoscopic Diagnostics and Therapeutics, Kyushu University Hospital, Higashi-Ku, Japan; 3 Department of Anatomic Pathology, Graduate School of Medical Sciences, Kyushu University, Higashi-Ku, Japan; 4 Department of Surgery and Science, Graduate School of Medical Sciences, Kyushu University, Higashi-Ku, Japan

**Correction to: European Radiology**

10.1007/s00330-025-11765-0 published online 18 June 2025.

In the original article first and last names of all authors had been incorrectly assigned.

Furthermore author Nobuhiro Fujita’s name was misspelled as Fujuta Nobuhiro.

The original article has been corrected.

